# Hepatitis B virus genotypes among chronic hepatitis B patients reporting at Korle-Bu teaching hospital, Accra, Ghana

**DOI:** 10.11604/pamj.supp.2016.25.1.6170

**Published:** 2016-10-01

**Authors:** Anthony Zunuo Dongdem, Mawuli Dzodzomenyo, Richard Harry Asmah, Kofi Mensah Nyarko, Priscillia Nortey, Adwoa Agyei, David Nana Adjei, Ernest Kenu, Andrew Anthony Adjei

**Affiliations:** 1 Ghana Field Epidemiology and Laboratory Training Program, School of Public Health, College of Health Sciences, University of Ghana, Legon, Ghana; 2 Department of Epidemiology and Biostatistics, School of Public Health, University of Health and Allied Sciences, Ho, Volta Region, Ghana; 3 Department of Biological, Environmental and Occupational Health, School of Public Health, College of Health Sciences, University of Ghana, Legon, Ghana; 4 School of Biomedical and Allied Health Sciences, College of Health Sciences, Korle-Bu, Accra, Ghana; 5 Department of Medicine, Korle-bu Teaching Hospital, Accra, Ghana; 6 Department of Pathology, University of Ghana Medical School, College of Health Sciences, Korle-Bu, Accra, Ghana

**Keywords:** Hepatitis B Virus, hepatitis B genotype, chronic hepatitis B carriers

## Abstract

**Introduction:**

Knowledge of hepatitis B virus (HBV) genotype is an important predictive variable which might have an impact in management and treatment of patients with chronic hepatitis B infection. In Ghana very little information is available on hepatitis B genotypes. This study was conducted to determine the distribution of HBV genotypes circulating among chronic hepatitis B patients reporting at the Korle-Bu Teaching Hospital (KBTH), Accra, Ghana.

**Methods:**

Blood samples (10 ml) were collected from 250 consenting patients. DNA was extracted and amplified using polymerase chain reaction technique. Restriction fragment length polymorphism (RFLP) was used for the detection of genotypes.

**Results:**

Out of the 250 chronic hepatitis B patients who were HBsAg positive, 91 (36.4%) were males aged 29.8 ± 9.1 and 159 (63.6%) females aged 33± 12.1 years. HBV DNA was detected in 111 (44.4%) but only 58 (52%) of these were typeable. These were classified as genotype A, 8 (7.2%); genotype D, 3 (2.7%) and genotype E, 47 (42.3%). Our results did not show any association between the infecting genotype and age (X^2^= 0.923; p-value=0.623) or gender (X^2^= 0.283, p= 0.579).

**Conclusion:**

Consistent with similar studies worldwide, the results suggest that genotypes A, D and E were the genotypes circulating among chronic hepatitis B patients who reported to the Korle-Bu Teaching Hospital with genotype E being the most predominant and therefore constitutes an important public health concern. We recommend further epidemiological studies to understand the implication of genotype E in terms of disease progression and treatment.

## Introduction

Hepatitis B virus is a DNA virus and the most common among the hepatitis viruses that cause chronic infections in humans. About two billion people globally have been exposed to HBV with approximately 5% of the world’s populations (350 million people) being chronic carriers [1], of which about 500,000-1.2 million hepatocellular carcinoma (HCC) associated deaths occur annually[2, 3]. HBV is classified into eight genotypes (A to H) based on a sequence divergence of more than 8% throughout the genome [4]. Two additional genotypes (I and J) have recently been proposed in Asia [5, 6]. The diversity among the genotypes has led to the division of some genotypes into sub-genotypes [7]. Each of these genotypes has a distinct geographical distribution between and within regions [8]. For instance genotype A is predominant in Sub-Saharan Africa, genotypes B and C in South East Asia, genotype D in Africa and Mediterranean countries, genotype E in Western and Central Africa, genotypes F and H in Central and South America, genotype G in North America [8–10].

HBV infection is emerging in Ghana and autopsy reports have revealed HBV infection to be a major risk factor for developing liver cirrhosis and HCC [11]. Knowledge about the infecting HBV genotypes has proven useful in understanding disease progression and response to antiviral treatment [12]. In Ghana, very little information is available on the circulating HBV genotypes. Chronic hepatitis B patients who meet the treatment criteria are being treated with variable response. The aim of this study was to determine the circulating HBV genotypes among chronic hepatitis B patients reporting at the Korle-Bu Teaching Hospital (KBTH), Accra, Ghana. Information from this current study will help in understanding the molecular epidemiology of the virus and possibly provide guidance to clinicians in the management of chronic hepatitis B patients in future.

## Methods

### Study design

This cross sectional study was carried out among chronic hepatitis B patients reporting at the KBTH from October 2009 to June 2010.

### Study site description

The study was conducted at the Gastroenterology Unit and the Central Laboratory of the KBTH, Accra, Ghana. The KBTH is situated in the nation’s capital, Accra, and it is the leading tertiary hospital and a major referral centre in the country. The Gastroenterology Unit is the biggest tertiary care for hepatitis cases in the country. More than 70% of the total cases in the country receive care and treatment at the Unit.

### Study population

The study subjects were chronic hepatitis B patients. Chronicity of the patients was determined based on their hepatitis B profile data (HBcIgM-negative and HBcIgG-positive). Patients on antiviral therapy for hepatitis B infection and those reporting on repeated visits were excluded from the study. A structured questionnaire was used to obtain socio-demographic characteristics of the subjects after which a blood sample was collected from each participant.

### Ethical clearance

Permission was sought from the KBTH authorities and approval granted by the Research and Ethical Review Committee of the Ghana Health Service. Informed consent was also obtained from patients or guardian of the patients before enrollment into the study. Patient’s confidentiality was assured by allocating identity numbers.

### Sample collection and storage

Blood samples (10ml) were collected from each of the consenting patients in EDTA tubes. Samples were centrifuged and serum kept at -20˚C until analyzed.

### Laboratory analysis

#### Genomic DNA extraction

Total DNA was extracted from plasma of all HBsAg positive kit using the QIAamp DNA Mini kit (Qiagen Co. Ltd UK) in accordance with the manufacturer’s instructions.

### Polymerase chain reaction (PCR) analysis

PCR detection of HBV DNA used was based on the protocol described previously [13]. The primers used, 5`GTGGTGGACTTCTCTCAATTTTC 3` (forward) and 5`CGGTATAAAGGGACTCACGAT 3` (reverse), flank specifically the S region of the HBV genome. PCR core kit (BioPioneers) was used. PCR was carried out in 25 µl reaction volumes containing 2.0 µl of 10X PCR buffer, 1.5 µl of 25 mM MgCl2, 0.5 µl of 10 mM each of deoxyribonucleoside triphosphate (dNTPs), 0.5 µl of 10 mM of each oligonucleotide primer (Sigma- Aldrich., USA) 0.5 U of DNA Taq polymerase enzyme (Sigma- Aldrich., USA). Ten microlitres (10 µl) of extracted DNA was used as template. Each reaction was thoroughly mixed by vortexing and flush centrifuged. The conditions used in the PCR amplification were initial denaturation at 94˚C for 3 minutes, followed by 40 cycles of denaturation at 94˚C for 45 seconds, annealing at 53˚C for 60 seconds and extension at 72˚C for 90 seconds. This was then followed by one cycle of 72˚C for 7 minutes [13] using a GeneAmp PCR System 2400 thermal cycler (Perkin Elmer, Norwalk CA, USA). The presence of HBV DNA was detected by running the PCR product on 2% agarose gel containing 0.5 µg/ml ethidium bromide.

### Restriction fragment length polymorphism (RFLP) analysis

PCR positive products obtained were doubly digested with Hinfl and Tsp5091 (New England BioLabs) restriction enzymes. The HinfI reaction had a total digestion reaction of 15.0 µl containing1.5 µl of X10 buffer, 3.0 µl of nuclease-free water, 0.50 µl of the enzymes and 10 µl of the PCR product. This was incubated at 37˚C for three hours. The Tsp509I reaction also had a total reaction volume of 15.0 µl containing10 µl of PCR products, 1.5 µl of X10 buffer, 2.5 µl of nuclease free water and 1.0 µl of the enzyme. This was also incubated at 65˚C for 3 hours. Ten microlitres of PCR product was mixed with1.5 µl of X10 buffer and 3.5 µl of nuclease free water but no restriction enzymes were run parallel with the enzyme treated sample as controls. The reactions were terminated by adding 4 µl of the loading buffer. A 3% agarose gel containing 0.5 µg/ml ethidium bromide was used to separate the restriction fragment length polymorphism (RFLP) products.

### Statistical analysis

Data was entered in Excel and statistical analysis was carried out using STATA (version 10.0) statistical packages. For socio demographic categorical data (e.g sex), summary tables of counts and percentages were presented. For socio demographic continuous data (e.g. age), summary tables of means, standard deviations were presented. Pearson’s chi square test and Fisher’s exact test were used to establish associations between demographic data and various outcome variables. All statistical tests were conducted as two-sided and declared significant for p-value < 0.05.

## Results

### Characteristic of the subjects

**Figure 1:**
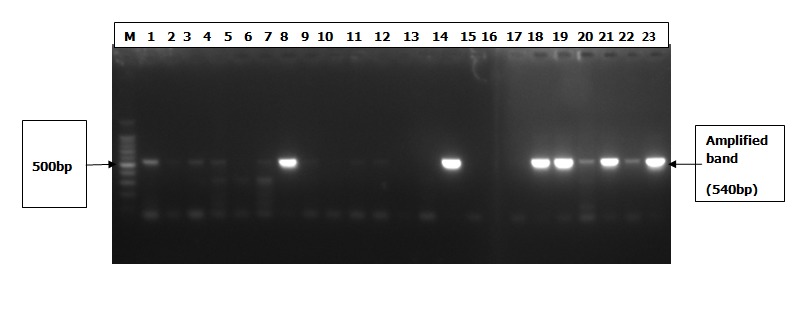
Shows gel electrophoresis pattern for HBV PCR positive samples. Lane M, 100bp molecular base marker, lanes 1-23 are with HBV DNA [540bp]

Two hundred and fifty (250) chronic hepatitis B patients were enrolled. The participants were 91 (36.4%) males aged 33±12.1 years and 159 (63.6%) females aged 29.3±9.1 years. (Table 1) shows HBV DNA detection by age and sex in the studied population. HBV DNA was detected in 111 (44.4%) of the 250 patients screened by PCR. We found that more males 44 out of 91 (48.4%) were frequently infected than females 67 out of 159 (42.1%). This was however not significant (X^2^=0.905; p-value=0.341). We also found that HBV infection to be highest (60%) in both the younger age group (10 - 19 years) and in the older age group of greater than 59 years and the lowest was found in age group 50 - 59 years. These differences were not significant (X^2^=5.35; p-value=0.500). (Figure 1) shows the gel electrophoresis pattern for HBV PCR positive samples.

**Table 1:** HBV DNA detection by age and sex among chronic hepatitis B patients

	HBV DNA			
Characteristics	Positive	Negative	Total	
**Age**				
0-9	2 (40.0)	3 (60.0)	5 (100)	
10-19	6 (60.0)	4 (40.0)	10 (100)	
20-29	47 (43.9)	60 (56.1)	64 (100)	X^2^ =5.35 (p-value=0.500)
30-39	44 (47.3)	49 (52.7)	93 (100)	
40-49	7 (36.8)	12 (63.2)	19 (100)	
50-59	2 (18.2)	9 (81.8)	11 (100)	
>59	3 (60.0)	2 (40.0)	5 (100)	
**Sex**				
**Male**	44 (48.4)	47 (51.6)	91 (100)	X^2^ = 0.905 (p-value= 0.341)
**Female**	67 (42.1)	92 (57.9)	159 (100)	
**Total**	111	139	250	

* figures in parenthesis represent percentages

### HBV genotype distribution

**Figure 2:**
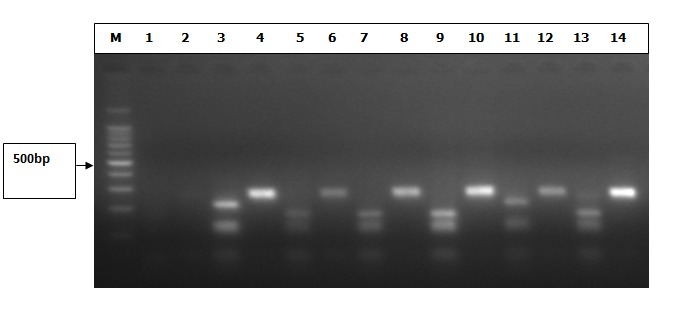
Shows the outcome of the RFLP of the S gene amplicon that was analyzed. Gel electrophoresis pattern for HBV genotypes obtained after restriction enzymes digest with Hinf 1 and Tsp 5091. Lane M, 100bp molecular base marker, the combination of lanes 4, 6, 8,10, 12 and 14 are one pattern (genotype A), the combination of lanes 3 and 11 are of one pattern (genotype D), the combination of lanes 5, 7, 9 and 13 are of another pattern (genotype E)

Out of a total of 111 samples that tested positive for HBV DNA only 58 (52.2%) showed genotype specific bands. The remaining 53 (47.7%) were untypeableas no genotype-specific band were seen. The distribution of the typeable genotypes is as follows: genotype A accounted for 8 (7.2%), genotype D, 3 (2.7%) genotype E, 47 (42.3%). The untypeable accounted for 53 (47.7%). The gel electrophoresis pattern for HBV genotypes obtained after restriction enzyme digest is shown (Figure 2). The predominant genotype of the study was E. The predominant genotype among males was E, 21(47.7%), followed by A, 4(9.1%). Similarly among females the predominant infecting genotype was E, 26(38.8%) followed by A 4(6.0%). Genotype D was the least typed in both sexes. Although there were differences in the proportion of the infecting genotype and gender of the patient, this was not significant (x^2^=0.283, p=0.579). Genotype A and E were predominant in the young patients ages between 20 - 29 years, and genotype D ages between (0 - 9 years), (20 - 29 years) and (30 - 39 years). However, genotypes were absent from individuals aged more than 59 years. This relationship between age and the infecting genotype was however found to be non-significant (X^2^=0.923; p-value=0.623). No mixed genotypes were found among the study subjects. The distribution of the HBV genotype by age and sex is shown (Table 2).

**Table 2:** Pattern of HBV genotype among chronic hepatitis B patients by age and sex (N=111)

	Genotype				
Age	A	D	E	Untypeable	
0-9	0 (0.0)	1 (33.3)	1 (2.1)	0 (0.0)	
10-19	0 (0.0)	0 (0.0)	5 (10.6)	1 (1.9)	
20-29	5 (62.5)	1 (33.3)	21 (44.7)	20 (37.7)	
30-39	2 (25.0)	1 (33.3)	17 (36.2)	24 (45.3)	X^2^=0.923 (p-value=0.623)
40-49	0 (0.0)	0 (0,0)	2 (4.3)	5 (9.4)	
50-59	1 (12.5)	0 (0.0)	1 (2.1)	3 (5.7)	
>59	0 (0.0)	0 (0.0)	0 (0.0)	0 (0.0)	
Sex					
Male	4 (9.1)	1 (2.3)	21 (47.7)	18 (40.9)	
Female	4 (6.0)	2 (3.0)	26 (38.8)	35 (52.2)	X^2^=0.283 (p-value=0.579)
Total	8 (7.2)	3 (2.7)	47 (42.3)	53 (47.7)	

* figures in parenthesis represent percentages

## Discussion

HBV is endemic in Ghana although there is scarcity of information on HBV genotypes. This study is the first conducted in Southern Ghana profiling the genotypic status of HBV among chronic hepatitis B patients in Ghana. The results of the study revealed that the circulating genotypes were A, D and E. This is in agreement with a study conducted among blood donors by Valente and colleagues in Angola who similarly reported the prevalence of genotypes A, D and E [14]. The findings are also in consonance with an earlier study in Ghana among blood donors at Komfo Anokye Teaching Hospital in Kumasi which similarly detected the prevalence of genotypes A, D and E [10]. These findings suggest that the genotypes present in blood donors are similar to those among chronic hepatitis B patients. In Africa, genotype A is prevalent in Southern Africa and genotype D in Northern Africa [15, 16]. In our study genotype E was found to be the most prevalent. This is consistent with other studies in West Africa which confirm genotype E predominance in West Africa [9, 10, 17, 18]. This findings further support the idea that genotype E is located in West Africa and common among African descendants [4, 9]. Many studies have revealed the severity and outcomes of disease to these genotypes; genotype C and D are thought to be more virulent in chronic carriers developing liver cirrhosis and HCC than genotypes A and B [19, 20]. It has also been reported by Flink and colleagues that genotype A response better to interferon alpha and pegylated interferon alpha than genotype D [21]. Thus the presence of these genotypes in our study population carries some important implication as regards disease progression and therapy. The clinical implication of genotype E has not been well studied [22, 23] although Fujiwara and colleagues speculated that HBV genotype E has a better prognosis than other genotypes in terms of liver disease [18].

In our study we could not detect minor subgenotypes and recombinant genotypes because the RFLP technique employed is less sensitive compared to the gold standard genome sequencing technique [24, 25]. This may account for the 47.7% of untypeables. Other studies using RFLP technique similarly reported untypeables [26]. These untypeables may represent novel, minor subgenotypes and recombinant genotypes in our study subjects. We also assessed the genotype distribution with respect to age and gender of patients. Genotype A and E were predominant in the younger patients less than 39 years and absent in the older age group (more than 59 years). This suggests that infections with these genotypes occur much earlier in life but contrasts the study in Pakistan among hepatitis B patients where no trend was found [26]. Our findings however agrees with a similar trend in a study in Japan, where genotype B presence increased from teenage to 50 years and genotype C, decreased from teenage to 50 years respectively [27]. The differences we detected were not significant and may only be a reflection of the age group in which majority of the samples were drawn. In terms of the infecting genotype and gender, genotype E was the most prevalent followed by genotype A in both males and females. These findings are in consonance with those of You et al among chronic HBV-infected patients in Yunnan, China [28] and contrasts the study by Yoshikawa who found genotype A mostly in male Japanese blood donors [27]. The study also demonstrated HBV DNA detection to be higher in males than females suggesting that more males with chronic hepatitis B were potentially more infectious than their female counterparts. Other studies have similarly suggested infections rates to be higher in males than females [29]. It is not clear how this happens but could be attributed to the chromosomal and endocrinal differences between males and females since males and females could have the same rate of exposure. In most HBV endemic Asian countries a lot of studies have been done to understand the molecular dynamics of HBV and its implication in the disease progression and management, our study is a step to determining the genotypes circulating among chronic hepatitis B patients, in Ghana. Future research needs be done in our country to better understand the molecular dynamics of HBV, since genotypes differ by geographical location [8, 10].

## Conclusion

Our study has shown the major circulating hepatitis B genotypes among chronic hepatitis B patients reporting to KBTH, Accra to be genotype A, D and E, with genotype E being the predominant. We recommend researchers to conduct similar studies using genome sequencing and further phylogenetic analysis to establish the existence of the minor/subgenotypes or recombinant or novel genotypes and also to determine the effect of these genotypes in the management of chronic hepatitis patients in Ghana.
